# Self‐Assembly of a Conjugate of Lipoic Acid With a Collagen‐Stimulating Pentapeptide Showing Cytocompatibility and Wound Healing Properties, and Chemical and Photolytic Disassembly

**DOI:** 10.1002/psc.70002

**Published:** 2025-02-04

**Authors:** Lucas R. de Mello, Valeria Castelletto, Leide Cavalcanti, Jani Seitsonen, Ian W. Hamley

**Affiliations:** ^1^ School of Chemistry, Food Biosciences and Pharmacy University of Reading, Whiteknights Reading UK; ^2^ ISIS Neutron & Muon Source, Science and Technology Facilities Council Rutherford Appleton Laboratory Harwell UK; ^3^ Nanomicroscopy Center Aalto University Espoo Finland

## Abstract

Lipoic acid is a biocompatible compound with antioxidant activity that is of considerable interest in cosmetic formulations, and the disulfide group in the N‐terminal ring confers redox activity. Here, we study the self‐assembly and aspects of the bioactivity of a lipopeptide (peptide amphiphile) comprising the KTTKS collagen‐stimulating pentapeptide sequence conjugated to an N‐terminal lipoic acid chain, lipoyl‐KTTKS. Using SAXS, SANS and cryo‐TEM, lipoyl‐KTTKS is found to form a population of curly fibrils (wormlike micelles) above a critical aggregation concentration. Upon chemical reduction, the fibrils (and β‐sheet structure) are disrupted because of the breaking of the disulfide bond, which produces dihydrolipoic acid. Lipoyl‐KTTKS also undergoes photo‐degradation in the presence of UV radiation. Through cell assays using fibroblasts, we found that lipoyl‐KTTKS has excellent cytocompatibility across a wide concentration range, stimulates collagen production, and enhances the rate of cell coverage in a simple in vitro scratch assay of ‘wound healing’. Lipoyl‐KTTKS thus has several notable properties that may be useful for the development of cosmetics, cell scaffolds or tissue engineering materials.

## Graphical abstract

The self‐assembly and bioactivity of the lipopeptide lipoyl‐KTTKS which comprises lipoic acid conjugated to a collagen‐stimulating pentapeptide are examined. Self‐assembly into curly fibrils (wormlike micelles) is observed, these structures being dissociated in the presence of a chemical reductant. The lipopeptide also undergoes photo‐degradation in the presence of UV radiation. Lipoyl‐KTTKS has excellent cytocompatibility, stimulates collagen production and enhances the rate of cell repopulation in a simple in vitro scratch assay.

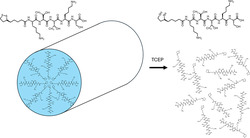

## 1 Introduction

Peptides are versatile, bioactive and biocompatible molecules, which are attracting interest for applications in biomedicine, tissue engineering and cosmetics among other applications. There are several classes of peptide for cosmetic applications including matrikines, which are short peptides derived by proteolysis of extracellular matrix components, which have cell signalling properties and are able to influence cell proliferation, migration and apoptosis [1, 2]. The KTTKS pentapeptide is used in a range of commercial anti‐wrinkle products, including the palmitoylated (C_16_‐hexadecyl‐linked) peptide C_16_‐KTTKS within the Matrixyl family. Lipidation is used to enhance in vivo stability (reducing degradation due to exopeptidase activity at the peptide terminal to which the lipid chain is attached) and potentially enhance skin permeability. Several reviews on cosmetic peptides that discuss this and related peptides are available [2, 3, 4, 5].

The KTTKS pentapeptide is based on a sequence taken from a pro‐peptide from human type I collagen [6, 7]. It has been reported that both the peptide KTTKS [6] and C_16_‐KTTKS [8] are able to promote type I collagen production in vitro. Studies also suggest that C_16_‐KTTKS can reduce the appearance of facial wrinkles [9, 10], although a recent critical article points to the lack of double blind and control experiments for clinical activity of matrikine peptides [11]. An important consideration in topical application of peptide amphiphiles is transport of the active type across the stratum corneum, and the lipidated conjugate was developed to improve delivery across the epidermis [9, 10, 12]. In prior work, the Hamley group has shown that C_16_‐KTTKS self‐assembles into highly extended nanotapes, based on a bilayer packing of the lipopeptide molecules [13]. We also investigated the self‐assembly of the related lipopeptides (peptide amphiphiles) C_14_‐KTTKS and C_18_‐KTTKS with distinct lipid chain lengths [14], as well as that of a lipopeptide with shorter peptide sequences C_16_‐KT (in comparison with other lipopeptides relevant to skincare applications) [15]. The pH‐dependent self‐assembly of C_16_‐KTTKS was also examined, and it was shown that it forms micelles at low pH in contrast to extended nanotapes at higher pH including neutral [16].

Lipoic acid is a biocompatible compound with antioxidant activity [17, 18, 19, 20], of considerable interest as a nutritional supplement and in cosmetic formulations. It has activity as a cofactor for mitochondrial enzymes in several important biological pathways [19, 21, 22, 23]. The disulfide group in lipoic acid confers redox activity, for example, the potential for intermolecular cross‐linking, which has been used, for example, to cross‐link vesicles containing lipoic acid phospholipids in the presence of dithiothreitol (DTT) [24]. The reduction of lipoic acid to dihydrolipoic acid occurs in mammalian cells and tissues [25, 26]. Glutathione (GSH) in cells can cleave disulfide bonds and GSH is overexpressed in cancer cells and redox‐response disulfide systems are of interest for cancer therapy [24].

**SCHEME 1:**
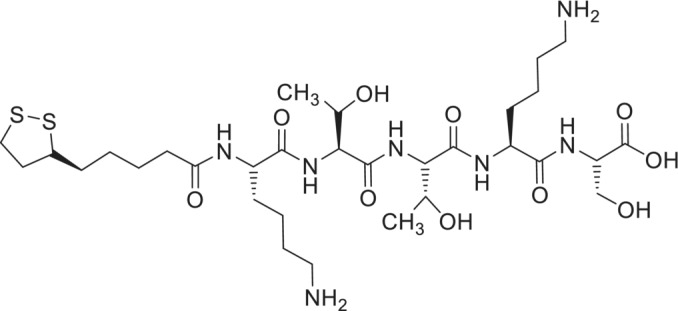
Molecular structure of lipoyl‐KTTKS.

Here, we investigate the structural properties, biocompatibility and collagen‐stimulating activity of a novel peptide amphiphile comprising a conjugate of lipoic acid to pentapeptide KTTKS, termed lipoyl‐KTTKS (Scheme 1). After starting our studies, we found that the synthesis of lipoyl‐KTTKS and outline biological studies have previously been reported [27]. In particular, this molecule shows promising antiaging properties related to UV‐induced damage, as it inhibits melanin synthesis, tyrosinase activity and UV‐induced collagenase (MMP‐1) activity whilst demonstrating good cytocompatibility and collagen synthesis superior to control and KTTKS itself. A lipoyl‐diethyleneglycol‐KTTKS conjugate showed similar properties [27]. Here, we investigate the self‐assembly of lipoyl‐KTTKS as well as the effect of chemical reduction on the nanostructure and UV‐induced photodegradation. The cytocompatibility of the compound to human dermal fibroblasts was examined along with measurements of collagen production. The inflammatory (or anti‐inflammatory) activity of the lipopeptide was examined using a Griess assay of nitrite ions, an in vivo modulator of inflammatory responses. As a simple model for wound healing, cell scratch assays were also performed.

## 2 Experimental

### 2.1 Materials

The peptide was obtained from Peptide Synthetics (Peptide Protein Research), Farnham, UK, as TFA salt with > 95% purity as confirmed by RP‐HPLC. The molar mass by ESI‐MS is 751.94 g mol^−1^ (751.36 g mol^−1^ expected). Adult Human Dermal Fibroblasts (HDFa) cells were obtained from Sigma‐Aldrich (UK, product code 106‐05A) and Dulbecco's Modified Eagle Medium (DMEM), DMEM/F12, Glutamax, penicillin–streptomycin and foetal bovine serum (FBS) were obtained from Thermo Fisher Scientific, UK. Direct Red 80 (Sirius red) and 3‐[4,5‐dimethylthiazol‐2‐yl]‐2,5‐diphenyltetrazolium bromide (MTT) were obtained from Sigma‐Aldrich. RAW 264.7 cells were obtained from ATCC and kindly donated by Dr. Bruno Mendes and Prof. Glyn Barrett. Griess reagent was obtained from Thermo Fisher Scientific, UK. The *Escherichia coli* lipopolysaccharide (LPS) was purchased from Sigma‐Aldrich (UK).

### 2.2 Circular Dichroism (CD) Spectroscopy

Far‐UV CD spectra were collected using a Chirascan spectropolarimeter (Applied Photophysics, Leatherhead, UK) equipped with a thermal controller. Spectra were recorded from 180 to 400 nm. Samples were mounted in a quartz cell with detachable windows, with 0.01‐nm path length. CD signal from the samples was corrected by water background subtraction. Where necessary, the CD signal was smoothed using the Chirascan Software for data analysis. The residual was chosen to oscillate around the average, to avoid artefacts in the smoothed curve. CD data, measured in mdeg, was normalized to molar ellipticity using the molar concentration of the sample and the cell path length.

### 2.3 Fourier Transform Infrared (FTIR) Spectroscopy

FTIR spectra were obtained using a Thermo‐Scientific Nicolet iS5 instrument with a DTGS detector. The solution was placed in a Specac Pearl liquid cell with CaF_2_ plates. For each sample, a total of 128 scans were recorded over the range of 900–4000 cm^−1^. For the range 450–1000 cm^−1^, a Perkin‐Elmer Spectrum Two FT‐IR instrument was used to collect spectra from UV irradiated peptide powder deposited on a diamond crystal plate, with 128 accumulated scans.

### 2.4 UV Exposure Studies

To investigate the effect of UV exposure on lipoyl‐KTTKS the peptide powder was irradiated using a 4W UVGL‐25 lamp (Analytik Jena, Germany) at a wavelength of 365 nm for 1 h. The absorbance was tracked at 333 nm before, just after exposure and 24 h after exposure using a nanodrop spectrometer (Thermo‐Fisher Scientific). The full spectra were also collected using a Varian Cary Bio 300 UV‐vis (Cary).

### 2.5 Small‐Angle X‐Ray Scattering Experiments (SAXS)

SAXS experiments were performed on beamline B21 [28] at Diamond (Didcot, UK). The sample solution was loaded into the 96‐well plate of an EMBL BioSAXS robot and then injected via an automated sample exchanger into a quartz capillary (1.8‐mm internal diameter) in the X‐ray beam. The quartz capillary was enclosed in a vacuum chamber, to avoid parasitic scattering. After the sample was injected into the capillary and reached the X‐ray beam, the flow was stopped during the SAXS data acquisition. Beamline B21 was operated with a fixed camera length (3.9 m) and fixed energy (12.4 keV). The images were captured using a PILATUS 2M detector. Data processing was performed using dedicated beamline software ScÅtter.

### 2.6 Small‐Angle Neutron Scattering (SANS)

Measurements were performed using the LOQ instrument at ISIS Neutron and Muon Source, Oxfordshire, UK. LOQ operates in time‐of‐flight mode, with a wavelength range from 2.7 to 10.5 Å. Two detectors recorded simultaneously small and wide scattering merged in a typical range of wavenumbers *q* = 0.006 Å^−1^ to 1.2 Å^−1^. The main detector was fixed at 4 m from the sample, and the size of the beam was 8 mm diameter at the sample position. The samples were placed in quartz cuvettes and arranged on a multislot sample changer with temperature controlled by a thermal bath at 25°C. The background was subtracted and the scattering intensity normalized to the absolute scale cross calibrating with a polystyrene standard. Data reduction was performed using Mantid [29].

### 2.7 Cell Culture

For the cytocompatibility assays, HDFa cells were cultivated in DMEM/F12 supplemented with 5% FBS, Glutamax, penicillin/streptomycin 100 IU/mL and incubated at 37°C within an atmosphere of 5% CO_2_. The RAW cells were cultured in DMEM with 10% FBS, Glutamax, penicillin/streptomycin 100 IU/mL and maintained under the same conditions as the HDFa cells.

### 2.8 MTT Assays

To measure the cytocompatibility of lipoyl‐KTTKS, 24 h before the assay HDFa cells were cultivated in 96‐well plates with 1 × 10^4^ cells/well with supplemented DMEM/F12. Then, the wells were washed three times with PBS, and cells were incubated for 72 h in media containing different concentrations of lipoyl‐KTTKS. After incubation, the cells were washed again three times with PBS and 100 μL of DMEM/F12 without phenol red + 5 μg/mL of MTT was added to each well. The plate containing cells in media containing MTT was incubated for 4 h inside an incubator at 37°C, protected from the light. After incubation, 100 μL of DMSO was added to each well to solubilize the resulting formazan crystals. The absorbance was measured at 560 nm using an Infinite F50 microplate reader (TECAN, Switzerland).

### 2.9 Collagen Quantification

To quantify collagen production, HDFa cells were seeded at a confluency of 1 × 10^4^ cells/well into 96‐well plates in DMEM/F12 + 5 μg/mL of insulin and incubated for 3 days in DMEM/F12 without supplements as control, or DMEM/F12 + different concentrations of lipoyl‐KTTKS and conventional supplements (ascorbic acid 1 mM, TGFβ_1_ 0.025 ng/mL and insulin 10 μg/mL). After incubation, the cells were washed three times with PBS and were fixed with cold ethanol 70%. The plate was transferred to a −80°C ultra‐freezer for 10 min and washed with water to remove any residual ethanol. After fixation, the collagen was incubated overnight in a solution containing Direct red 1 mg/mL in picric acid at 4°C. After this final incubation, the fixed cells were washed with distilled water to remove any residual dye, and the collagen and cells were then treated with 1‐mM NaOH for 10 min at room temperature. The resulting absorbance was measured at 490 nm using an Infinite F50 microplate reader (TECAN, Switzerland) [8].

### 2.10 Nitrite (Inflammation) Assay

To detect nitrite, the Griess reagent assay was used, following protocols described previously [30, 31]. Amounts 5 × 10^3^ RAW 264.7 cells were seeded in a 96‐well plate and incubated for 24 h inside the cell incubator. After 24 h, the cells were washed three times with PBS and incubated in 100 μL of DMEM + 2 μg of LPS (lipopolysaccharide) for 24 h. After incubation in the presence of LPS, 100 μL of Griess reagent solution was added to each well and incubated for 10 min at room temperature. The resulting absorbance was measured at 550 nm using an Infinite F50 microplate reader (TECAN, Switzerland).

### 2.11 Scratch Assay

For the scratch assay, 5 × 10^4^ cells into were seeded into 24‐well plates, and cells were allowed to reach confluence. After reaching 90% confluence, a scratch was made in the middle of the well using a 200‐μL pipette tip and cells were incubated at 37°C in an atmosphere 5% CO_2_. The media used was DMEM without serum for the controls or DMEM without serum + lipoyl‐KTTKS at different concentrations. Pictures were taken periodically using a standard inverted optical microscope with phase contrast filters to monitor cell migration and wound closure.

## 3 Results

**FIGURE 1:**
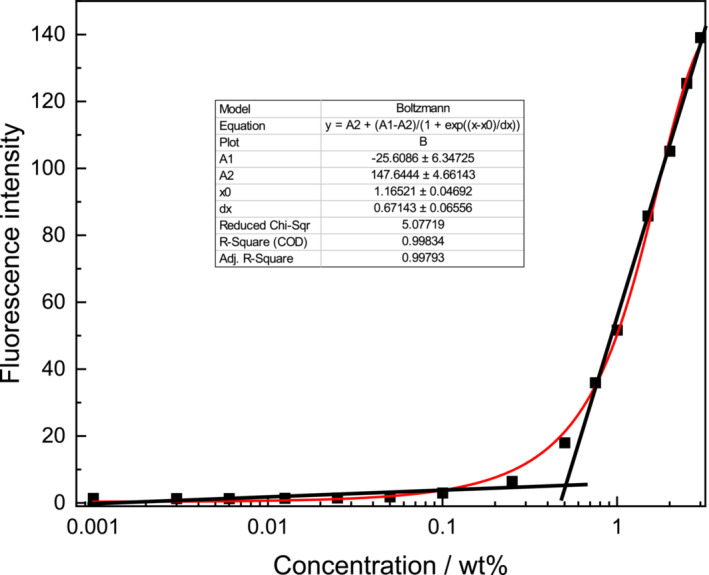
CAC assay plotting fluorescence intensity of ThT (*λ*_em_ = 448 nm) as a function of concentration. The inset shows the fit parameters for the Boltzmann‐type sigmoidal function fit (red line). The black lines provide a linear intercept estimate of CAC.

Before considering biocompatibility and bioactivity of lipoyl‐KTTKS, we examined whether this lipopeptide has a critical aggregation concentration (CAC) above which it forms self‐assembled aggregate structures. Based on our previous work on KTTKS‐lipopeptides [13, 14, 15], β‐sheet formation of lipoyl‐KTTKS is likely to be favoured. Therefore a CAC assay using the fluorescent probe thioflavin T (ThT) which is sensitive to β‐sheet formation [32, 33] was performed. The ThT fluorescence peak intensity was monitored as a function of concentration, and the results are shown in Figure 1 and indicate a CAC value (0.5 ± 0.1) wt% from the intersection of the straight lines at low and high concentrations, or (0.67 ± 0.06) wt% as the midpoint of a fitted Boltzmann‐type sigmoidal function [34, 35]:

$$I=A_{2}+\frac{A_{1}-A_{2}}{1+\exp\left[\frac{c-c_{1/2}}{\sigma }\right]}$$

Here, *I* is the fluorescence intensity (at *λ*_em_ = 448 nm), *A*_1_ and *A*_2_ are constants, *c* is the lipopeptide concentration, *c*_1/2_ is the mid‐point concentration, and *σ* is the width parameter.

The CAC values obtained from the two analysis methods are consistent and indicate a relatively high CAC value for lipoyl‐KTTKS compared with C_16_‐KTTKS [8, 15] and related molecules such as C_16_‐ETTES developed by the Hamley group [36].

**FIGURE 2:**
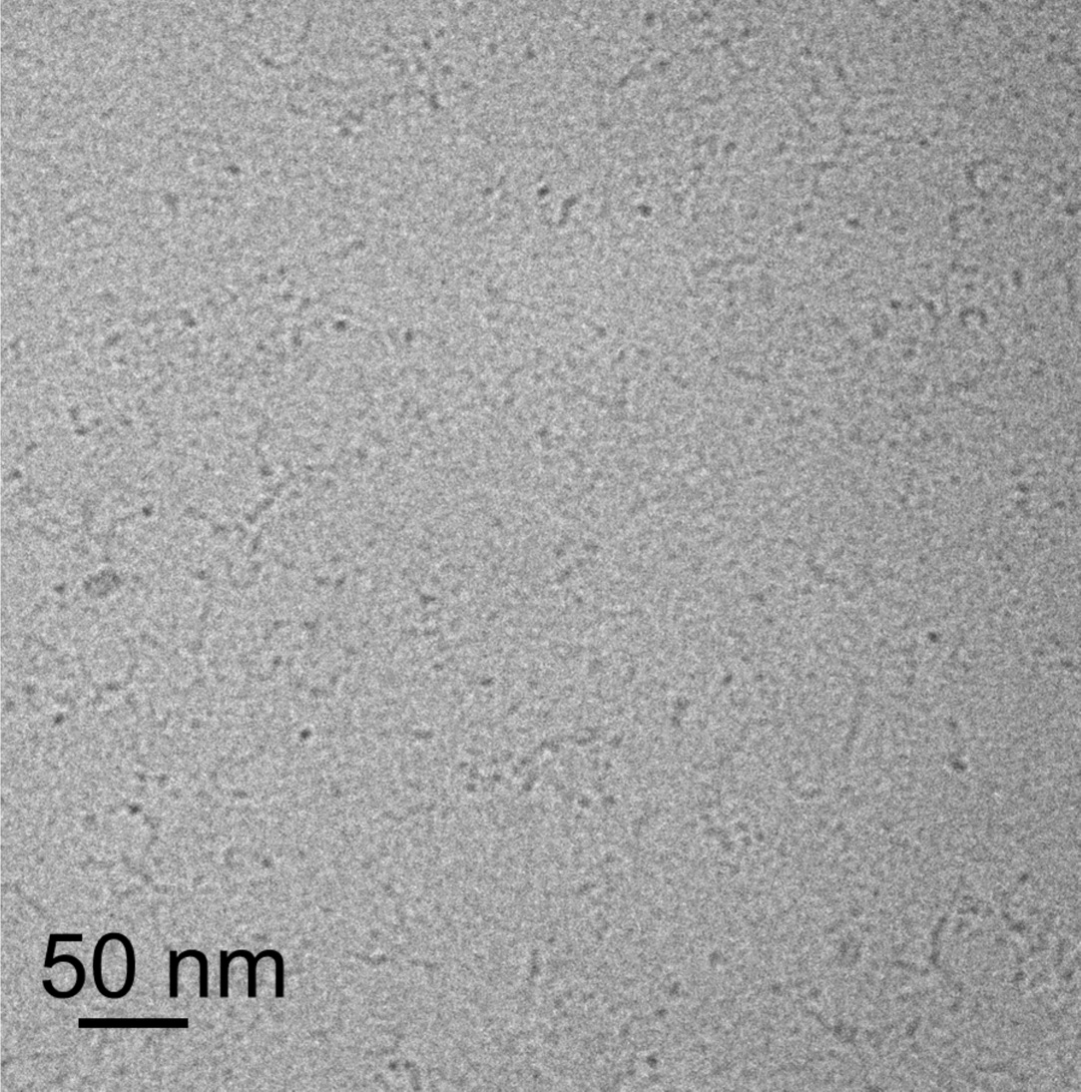
Cryo‐TEM image from a 3‐wt% aqueous solution of lipoyl‐KTTKS.

**FIGURE 3:**
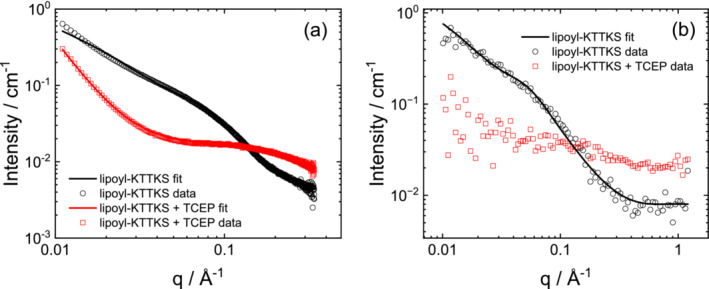
(a) SAXS and (b) SANS data for 3‐wt% lipoyl‐KTTKS in D_2_O in the absence or presence of 2.6‐wt% TCEP. The symbols are measured data (samples indicated) and the lines are form factor fits as described in the data (fit parameters in Table S1). For ease of visualization, only every fifth data point is plotted for the SAXS data.

Cryo‐TEM was used to probe potential aggregation in solution above the CAC (3 wt% aqueous solution). The cryo‐TEM image in Figure 2 shows the presence of short curly fibrils (extended oligomer structures). The presence of such structures was also confirmed by SAXS and SANS data (Figure 3), which reveal form factor features that can be fitted using a core‐shell cylinder form factor, consistent with extended fibril structures. The fit parameters are listed in Table S1. The cylinder radius is 25 Å, which is approximately equal to the estimated length of the molecule considering the lipid chain plus a pentapeptide in a β‐sheet conformation. The shell thickness 10 Å suggests that this only comprises the C‐terminal part of the peptide. For the SAXS data, it was necessary to include a contribution in the fits from unaggregated peptide, which gives rise to scattering at high *q*, described using a generalized Gaussian coil form factor (generalized refers to allowing the Flory exponent for the coil to vary from the value ν = 0.5 for an unperturbed coil [37, 38]). SANS data for 3 wt% solutions of lipoyl‐KTTKS in D_2_O can be fitted to a core‐shell cylinder form factor, consistent with SAXS, with fit parameters listed in Table S1. It was not necessary to allow for unaggregated monomer in fitting the SANS data, which may be because of the higher background and/or altered scattering contrast compared with SAXS. The cylinder radius and shell thickness obtained from the fits to the SANS data are in excellent agreement with the parameters obtained from the SAXS fit.

**FIGURE 4:**
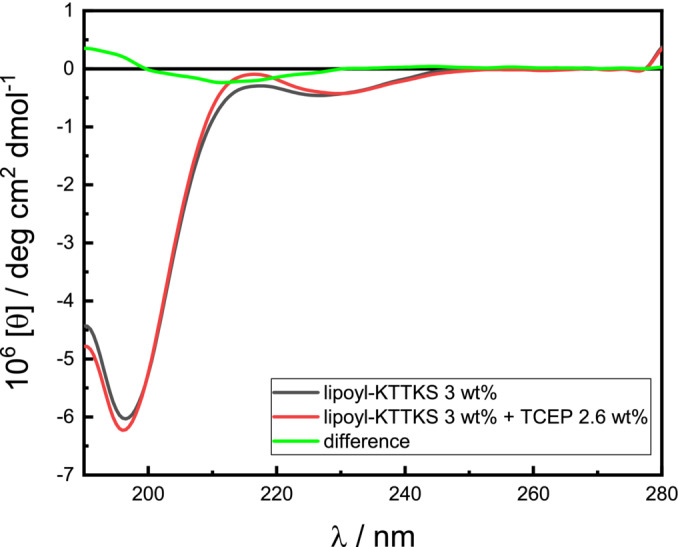
CD spectra for lipoyl‐KTTKS with and without TCEP and difference lipoyl‐KTTKS − (lipoyl‐KTTKS + TCEP).

**FIGURE 5:**
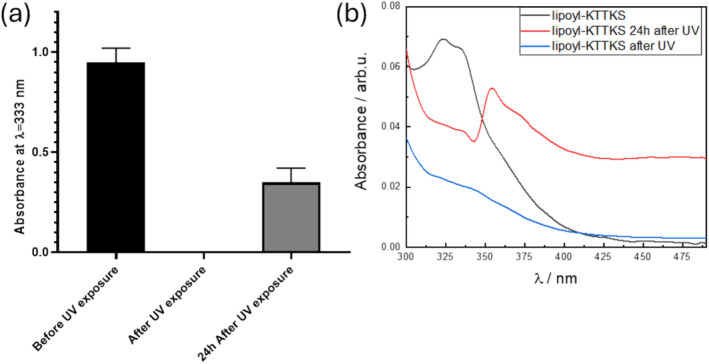
(a) UV‐vis absorption at 333 nm of lipoyl‐KTTKS before, just after irradiation and 24 h after irradiation (showing partial recovery). (b) Spectra for the same samples (as indicated).

The ring‐opening of the lipoyl moiety can be achieved under reductive conditions [24, 25, 26]. We examined the effect of chemical reduction on the self‐assembled structures of lipoyl‐KTTKS using TCEP [tris(2‐carboxyethyl)phosphine] as reductant. The SAXS and SANS data in Figure 3 show that the addition of TCEP disrupts the fibril structures. The SAXS data in Figure 3a show a considerable reduction in scattering intensity after addition of TCEP and the SANS profile in Figure 3b is barely above background after addition of TCEP. The SAXS data can be described using a form factor for monomer scattering with a power‐law intensity function at low *q* to account for a small fraction of irregular aggregates. The disruption of the fibrils upon TCEP addition was also confirmed by cryo‐TEM (images shown in Figure S1) because no aggregate structures were observed after addition of TCEP. Circular dichroism (CD) spectra shown in Figure 4 are characterized by a negative minimum in the CD spectrum at 196 nm along with the absence of defined features at higher wavelength, characteristic of a primarily disordered conformation [39, 40, 41]. However, taking the difference between the CD spectra for lipoyl‐KTTKS without and with TCEP produces a spectrum (green spectrum in Figure 5) with a minimum near 215 nm, characteristic of β‐sheet structure [39, 42, 43, 44]. This is consistent with the observation that lipoyl‐KTTKS forms fibril‐like structures above a CAC detected by ThT binding to β‐sheet structure and as observed by cryo‐TEM, SAXS and SANS.

Lipoic acid is susceptible to photolysis upon exposure to UV light [45, 46]. Photodegradation of lipoyl‐KTTKS was studied via UV‐vis, FTIR and CD spectroscopy and degradation products were analysed by electrospray ionisation‐mass spectroscopy (ESI‐MS). Photolysis was induced by exposure to UV for 1 h using a UV lamp with 365‐nm radiation. Lipoic acid presents a characteristic weak band at 333 nm that is related to the hindered dithiolane ring, that can be broken by UV irradiation [45, 47]. The absorbance of lipoyl‐KTTKS (0.1 wt% solution) before irradiation, just after and 24 h after irradiation to evaluate the recovery of the dithiolane ring is shown in Figure 5. There is incomplete recovery 24 h after UV exposure. The UV exposure disrupts the dithiolane ring, as reflected also in the FTIR spectrum (Figure S2), with the suppression of bands ν(S‐S) at 519 and 438 cm^−1^ and ν(C‐S) at 721 cm^−1^ [48, 49]. The photolysis produces complex mixture of degradation products, as revealed by ESI‐MS (Figure S4, compared with data for original peptide in Figure S3).

**FIGURE 6:**
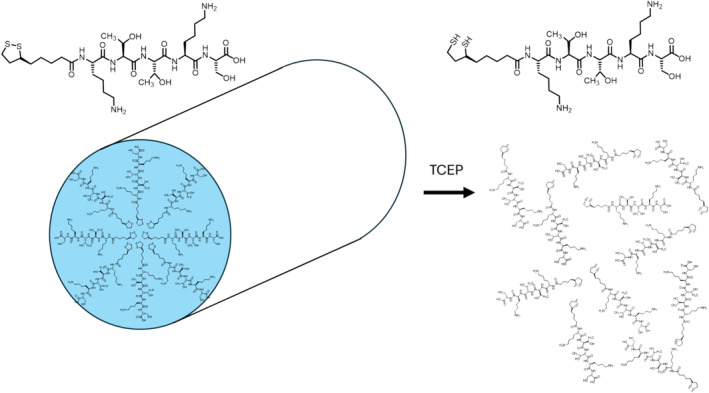
Showing TCEP reduction of lipoyl‐KTTKS to dihydrolipoyl‐KTTKS along with concomitant disassembly of lipoyl‐KTTKS fibrils.

The self‐assembly studies reveal, in summary, that lipoyl‐KTTKS forms a population of short curly fibrils (short wormlike micelles) above a CAC, with β‐sheet content as confirmed by ThT binding and CD spectra analysis. As well as fibrils, SAXS indicates the presence of a significant fraction of unassembled (monomeric) lipopeptide molecules (also indicated by the fact that the cryo‐TEM images are not fully populated with fibrils). The fibrils are destroyed upon reduction. This is summarized by Figure 6, which shows self‐assembled fibrils of lipoyl‐KTTKS (left) and molecularly dispersed dihydrolipoyl‐KTTKS after reduction (right). Lipoyl‐KTTKS also undergoes UV‐induced photodegradation.

Following on from the self‐assembly and conformational studies, we examined the bioactivity of lipoyl‐KTTKS in unreduced form. The cytocompatibility was first examined through measurement of cellular mitochondrial activity (MTT assays). We then examined the production of type I/III collagen along with a simple scratch assay of in vitro ‘wound healing’. We also probed anti‐inflammatory activity because of nitrite production upon exposure of cells to bacterial lipopolysaccharide (LPS), a common method to stimulate inflammation.

**FIGURE 7:**
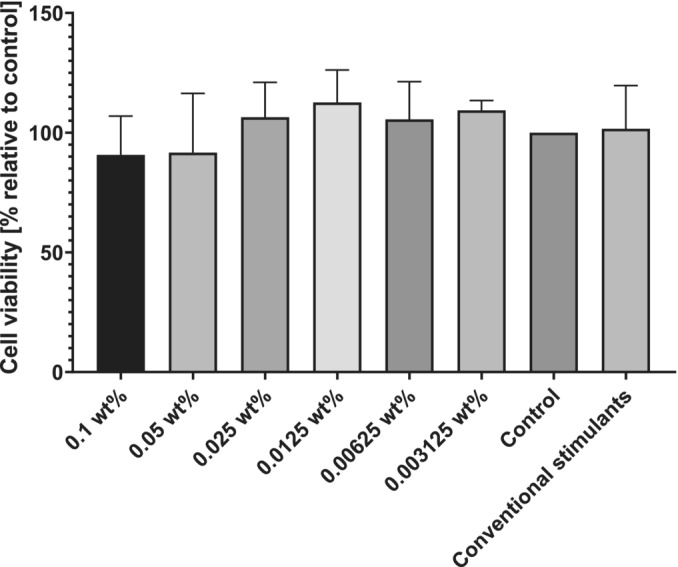
Cell viability from MTT assays for HDFa cells incubated for 72 h with different concentrations of lipoyl‐KTTKS, conventional stimulants (ascorbic acid 1 mM, TGFβ_1_ 0.025 ng/mL and insulin 10 μg/mL) or DMEM as a control. There were no statistically significant differences between the groups incubated with lipoyl‐KTTKS when compared with the controls via ANOVA, *n* = 3.

MTT assays were conducted to determine the cytocompatibility of this new molecule, and as indicated in Figure 7, lipoyl‐KTTKS does not present any statistically significant cytotoxicity even at higher concentrations, such as 0.1 wt%, as indicated by the ANOVA statistical test. This is promising for future applications in cell culture.

**FIGURE 8:**
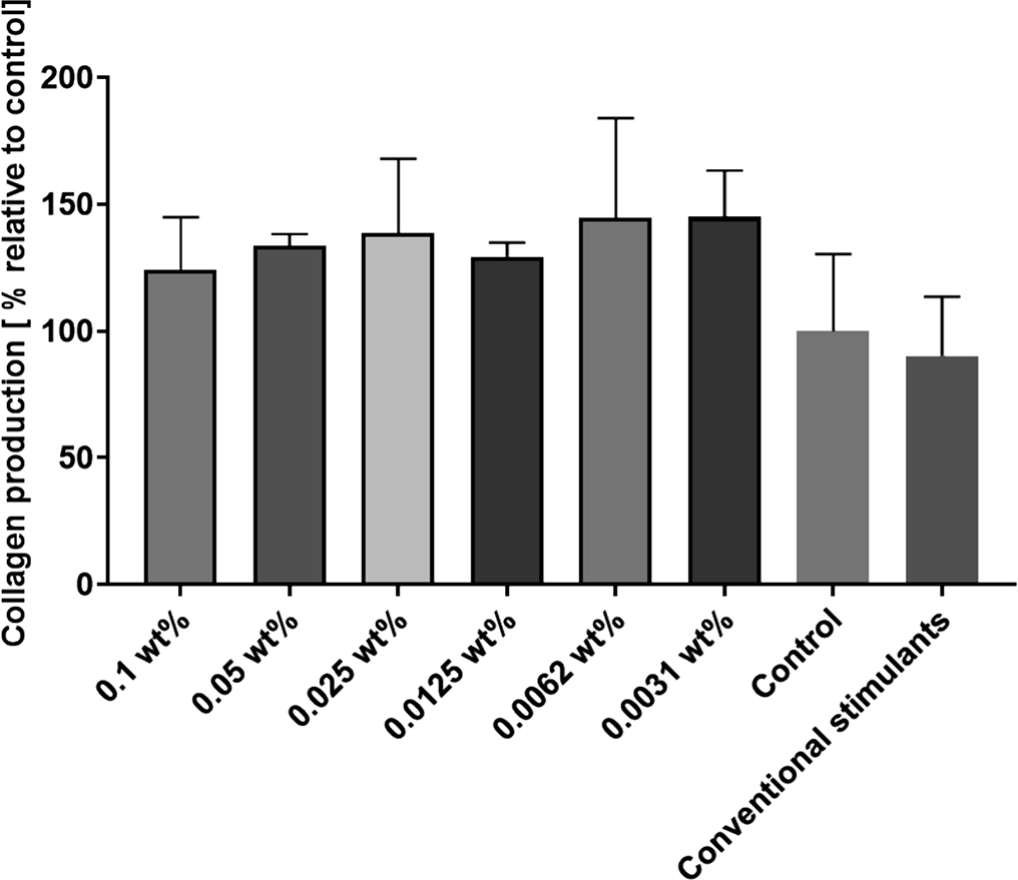
Collagen quantification assays performed using the picrosirius red technique. HDFa cells were incubated for 72 h with different concentrations of lipoyl‐KTTKS, conventional stimulants or just DMEM as a control. There were no statistically significant differences between the groups incubated with the lipopeptide when compared with the controls via ANOVA, *n* = 3.

Because of the great commercial interest in KTTKS pentapeptides in cosmetic products with proposed antiaging properties resulting from cellular matrix stimulation, collagen production assays were performed using the technique of picrosirius red (commercial name, Direct red 80) staining (UV/vis absorbance) measurements [50]. The results are shown in Figure 8, and although there was a visible increase in the production of collagen, the data show no statistically significant increase, when compared with the control or conventional stimulants. This can be contrasted with previous reported data for a 0.038 wt% (0.5 mM) sample of lipoyl‐KTTKS, which shows an apparent 65% increase in collagen synthesis compared with negative control [27]. However, this result is based on a different enzyme immunoassay of procollagen type I carboxy‐terminal peptide (PIP), not collagen itself, and it is not clear whether PIP is a good proxy for collagen in the case of studies using dermal fibroblasts.

As a simple in vitro ‘wound healing’ model, scratch assays were performed by creating a scratch in a layer of cells. Images of the confluent cell layer are presented in SI Figure S5. The scratch assay is a simple and affordable procedure consisting of simulating the closure of a skin wound by scratching a monolayer of fibroblasts seeded at high confluence and monitoring how long it takes for the cells to migrate and recover the ‘wound’ [51, 52, 53]. As evident from the images in Figure S5, the recovery of cells incubated with 0.0062 wt% lipoyl‐KTTKS was enhanced, with complete closure of the scratch after 48 h. This suggests an enhancement in cell migration compared with control cells incubated only with DMEM without serum, for which complete closure of the scratch was observed only after 72 h of incubation.

**FIGURE 9:**
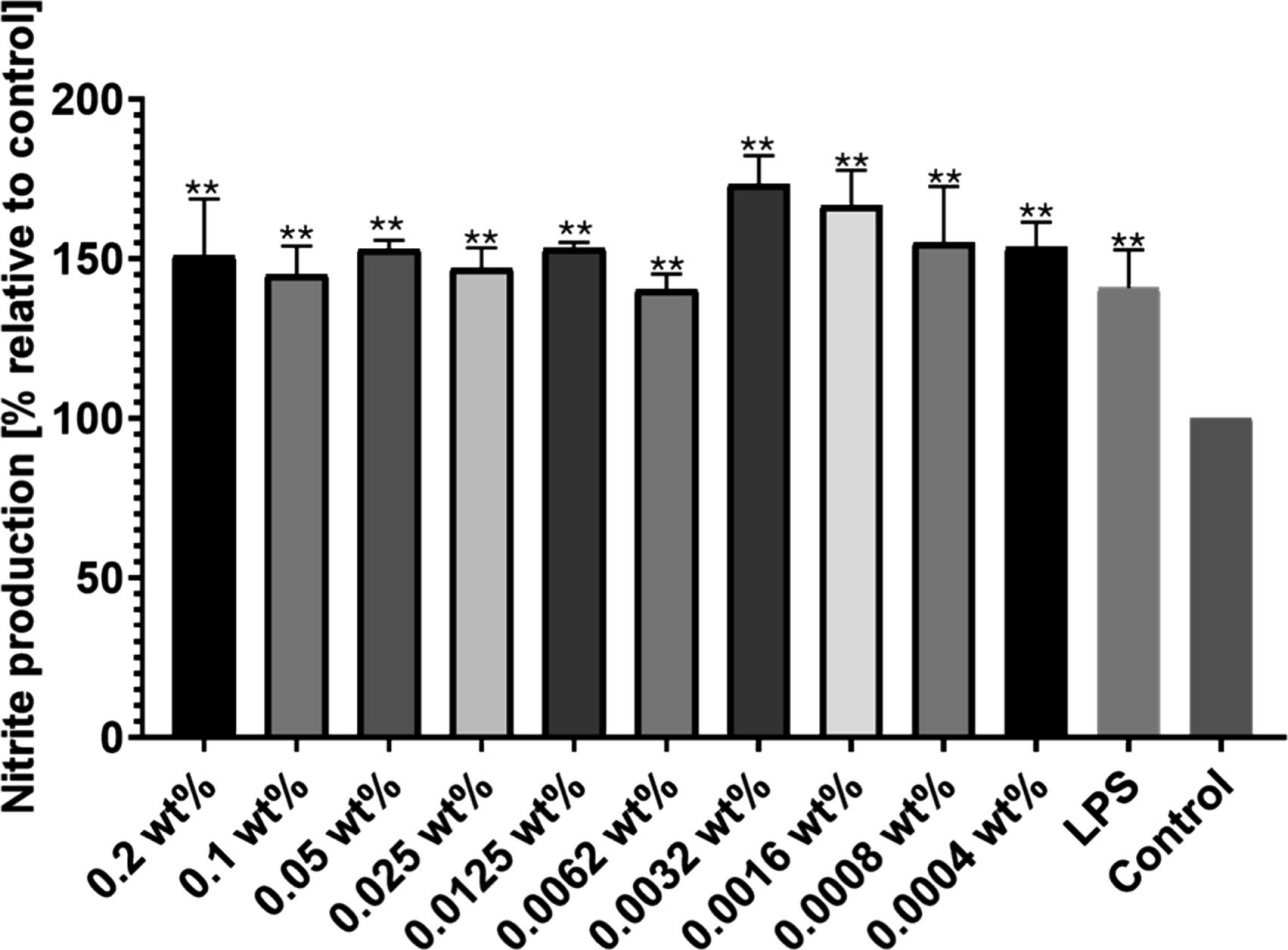
Nitrite production assay using Griess reagent for RAW 264.7 cells incubated with 2 μg of LPS for 24 h. All concentrations of lipoyl‐KTTKS studied presented statistically significant difference when compared with the control without LPS via ANOVA with Bonferroni correction for multiple comparisons, *n* = 3.

The Griess reagent assay was used as an indirect measure of the production of nitrite ions, an important species related to inflammation, by monitoring the presence of nitrites in media via colorimetric (UV‐vis absorbance) measurements. The assay was performed by incubating the cells in DMEM containing 2 μg of LPS with different concentrations of lipoyl‐KTTKS. Controls containing only LPS and RAW cells incubated in DMEM without LPS were also prepared. The ANOVA test with a Bonferroni correction for multiple comparisons demonstrated a statistically significant increase in the production of nitrites for RAW 264.7 cells incubated with the peptide and LPS when compared with cells incubated only in DMEM (Figure 9).

## 4 Discussion and Conclusions

Lipoyl‐KTTKS is shown to aggregate above a CAC detected by Thioflavin T dye fluorescence, a probe of β‐sheet structure. Using a combination of cryo‐TEM, SAXS and SANS, we show that lipoyl‐KTTKS forms a population of curly fibrils or wormlike micelles above the CAC, although there appears to be a significant fraction of monomer coexisting with the aggregates based on analysis of SAXS data and inspection of cryo‐TEM images such as Figure 2.

The lipopeptide disassembles in the presence of the chemical reductant TCEP. The molecule also shows photodegration in response to UV light. These redox properties may be useful for future applications such as cosmetics, indeed beneficial anti‐aging effects related to UV damage have already been noted [27].

Our findings from the MTT cell viability assay indicate that cells incubated for 72 h with different concentrations of lipoyl‐KTTKS maintained high levels of metabolic activity and are viable even when incubated with higher concentrations of this peptide. The lipopeptide stimulates collagen production, although not significantly greater than control. This is in contrast to previous findings (single data point reported at 0.038 wt%) [27], although as noted above this was based on a different enzyme immunoassay of procollagen type I carboxy‐terminal peptide (PIP), not collagen. However, we found positive bioactivity results from the scratch assay, because cells started showing signs of recovery after 12 h and the scratch completely recovered after 48 h in samples incubated with 0.0062 wt% lipopeptide compared with a longer period 72 h in samples incubated in basal media. Thus, lipoyl‐KTTKS promotes faster ‘wound healing’ in this simple in vitro assay.

The inflammatory nitrite assay using the Griess reagent indicated an increase in NO production by cells incubated with lipoyl‐KTKKS, with statistical significance for all concentrations of lipopeptide evaluated. Although an increase in nitric oxide is usually related to inflammation in skin, it can be useful in the case of conditions such as diabetes where the expression and production of NO is impaired. This leads to a reduction in keratinocyte numbers during re‐epithelization and slow clearance of pathogens in the case of infections, and an increase in NO would here be beneficial [54, 55]. In summary, lipoyl‐KTTKS shows interesting chemical and UV redox‐responsiveness and some positive bioactivities—high cytocompatibility and accelerated cell recovery following a scratch assay and increased nitrite production, of potential benefit to certain health conditions.

## Supporting information


**Figure S1** Cryo‐TEM images for (a) 1 wt% lipoyl‐KTTKS, (b) 1 wt% lipoyl‐KTTKS + 0.37 wt% TCEP.
**Figure S2.** FTIR spectra of 5 wt % samples of lipoyl‐KTTKS before (black) and after (red) UV exposition. Relevant vibrational bands are identified by the arrows.
**Figure S3.** ESI‐MS for lipoyl‐KTTKS before UV exposure.
**Figure S4.** ESI‐MS for lipoyl‐KTTKS after UV exposure, showing some starting peptide plus a range of degradation products with masses in the range 218–721 g mol^−1^.
**Figure S5.** Scratch assays of samples incubated in DMEM containing 0.0062 wt% of lipoyl‐KTTKS and only with DMEM without serum to evaluate the migration of HDFa cells and simulate wound recovery.
**Table S1.** SANS and SAXS data fitted parameters. Data fitted using SASfit,^[1, 2]^ using a long cylindrical shell form factor model, with allowance for monomers via a Generalized Gaussian coil factor for SAXS data.

## Data Availability

The data that support the findings of this study are available on request from the corresponding author. The data are not publicly available due to privacy or ethical restrictions.
