# Supplementary material for: Self‐Assembly of a Conjugate of Lipoic Acid With a Collagen‐Stimulating Pentapeptide Showing Cytocompatibility and Wound Healing Properties, and Chemical and Photolytic Disassembly
Source: J Pept Sci. 2025 Feb 4;31(3):e70002. doi: 10.1002/psc.70002 (PMC11794677; doi:10.1002/psc.70002)
Supplement: Supplementary file 1 — Figure S1 Cryo‐TEM images for (a) 1 wt% lipoyl‐KTTKS, (b) 1 wt% lipoyl‐KTTKS + 0.37 wt% TCEP. Figure S2. FTIR spectra of 5 wt % samples of lipoyl‐KTTKS before (black) and after (red) UV exposition. Relevant vibrational bands are identified by the arrows. Figure S3. ESI‐MS for lipoyl‐KTTKS before UV exposure. Figure S4. ESI‐MS for lipoyl‐KTTKS after UV exposure, showing some starting peptide plus a range of degradation products with masses in the range 218–721 g mol−1. Figure S5. Scratch assays of samples incubated in DMEM containing 0.0062 wt% of lipoyl‐KTTKS and only with DMEM without serum to evaluate the migration of HDFa cells and simulate wound recovery. Table S1. SANS and SAXS data fitted parameters. Data fitted using SASfit,[1, 2] using a long cylindrical shell form factor model, with allowance for monomers via a Generalized Gaussian coil factor for SAXS data. [file PSC-31-e70002-s001.docx]

**Supporting Information**

**Self-Assembly of a Conjugate of Lipoic Acid with a Collagen-Stimulating Pentapeptide Showing Cytocompatibility and Wound Healing Properties, and Chemical and Photolytic Disassembly**

Lucas R de Mello,^1^ Valeria Castelletto,^1^ Leide Cavalcanti,^2^ Jani Seitsonen,^3^ Ian W Hamley^1,*^

*^1^ School of Chemistry, Food Biosciences and Pharmacy, University of Reading, Whiteknights, Reading RG6 6AD, U.K.*

*^2^* *ISIS Neutron & Muon Source, Science and Technology Facilities Council, Rutherford Appleton Laboratory, Harwell, OX11 0QX, U.K.*

*^3^ Nanomicroscopy Center, Aalto University, Puumiehenkuja 2, FIN-02150 Espoo, Finland*

* Author for correspondence. I.W.Hamley@reading.ac.uk

**
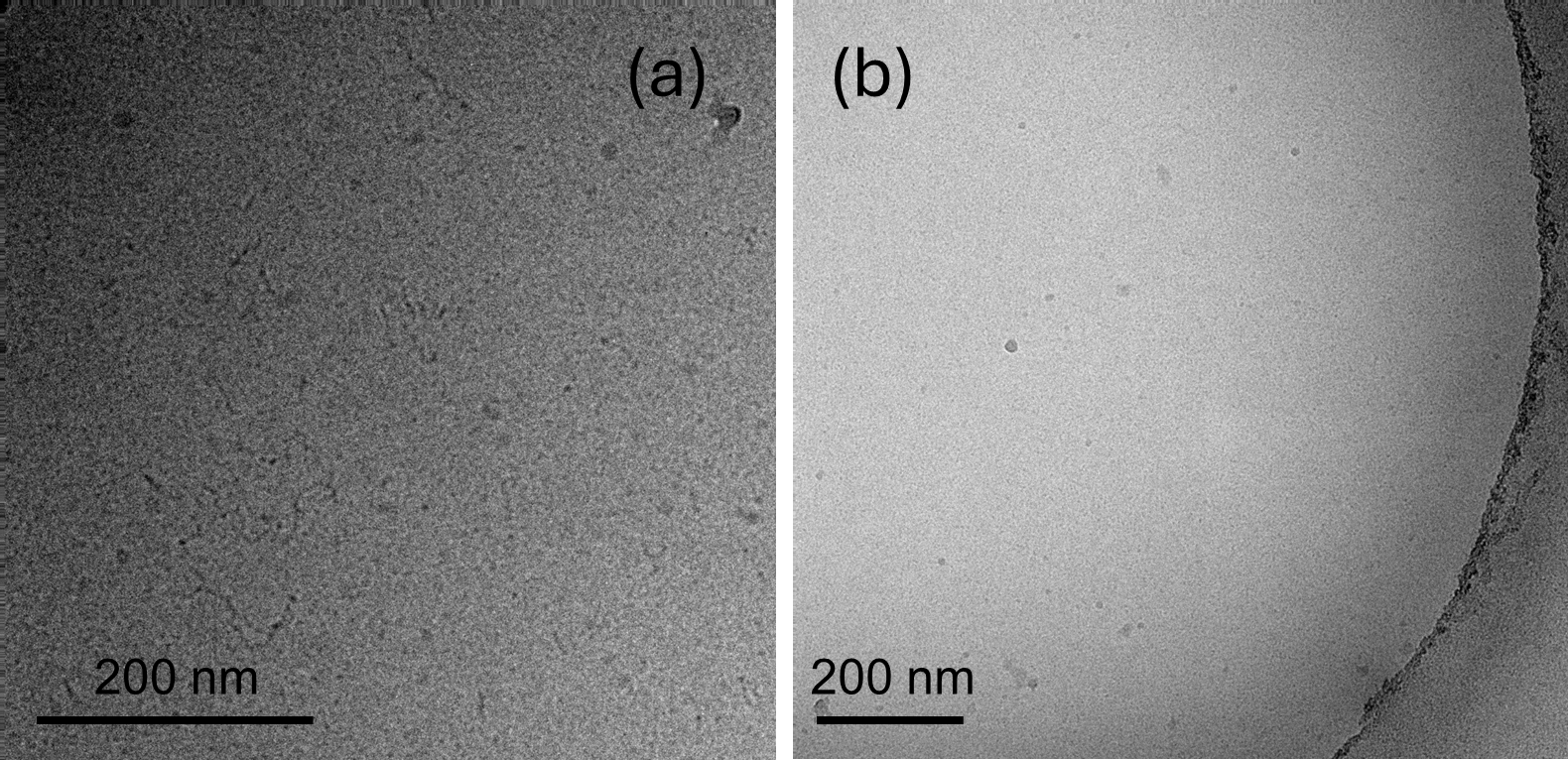
**

**Figure S1.** Cryo-TEM images for (a) 1 wt% lipoyl-KTTKS, (b) 1 wt% lipoyl-KTTKS + 0.37 wt% TCEP.


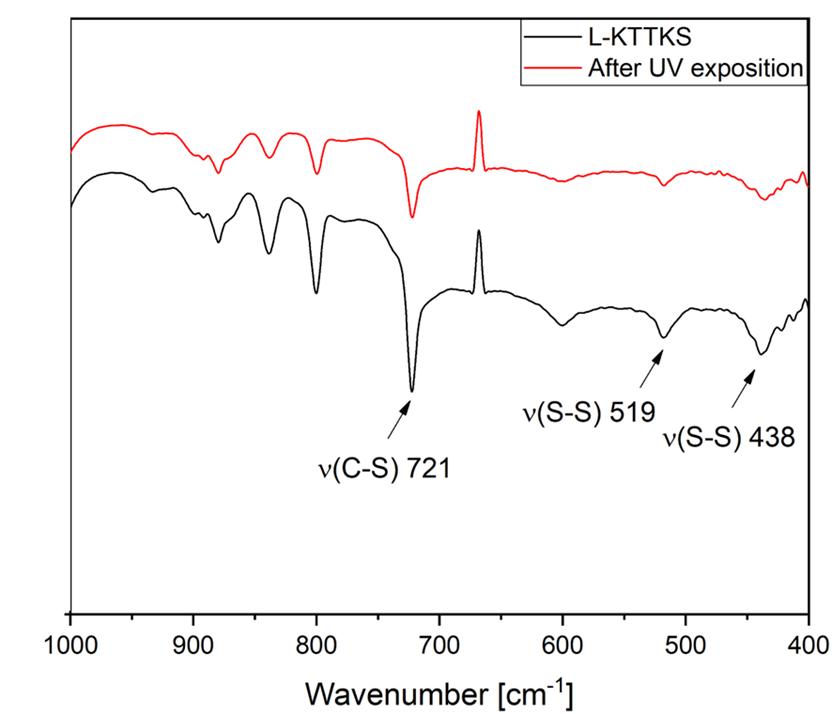


**Figure S2**. FTIR spectra of 5 wt % samples of lipoyl-KTTKS before (black) and after (red) UV exposition. Relevant vibrational bands are identified by the arrows.

**Figure S3**. ESI-MS for lipoyl-KTTKS before UV exposure.


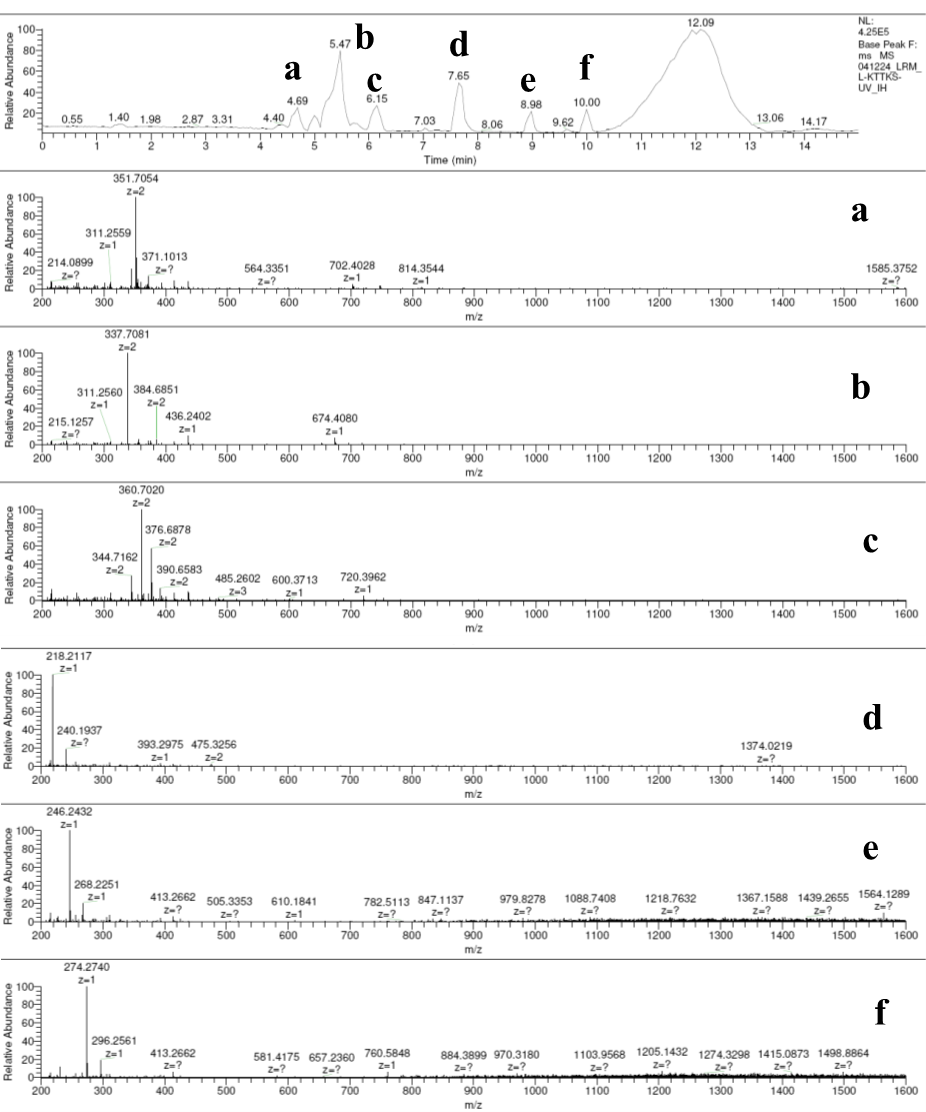


**Figure S4**. ESI-MS for lipoyl-KTTKS after UV exposure, showing some starting peptide plus a range of degradation products with masses in the range 218- 721 g mol^-1^


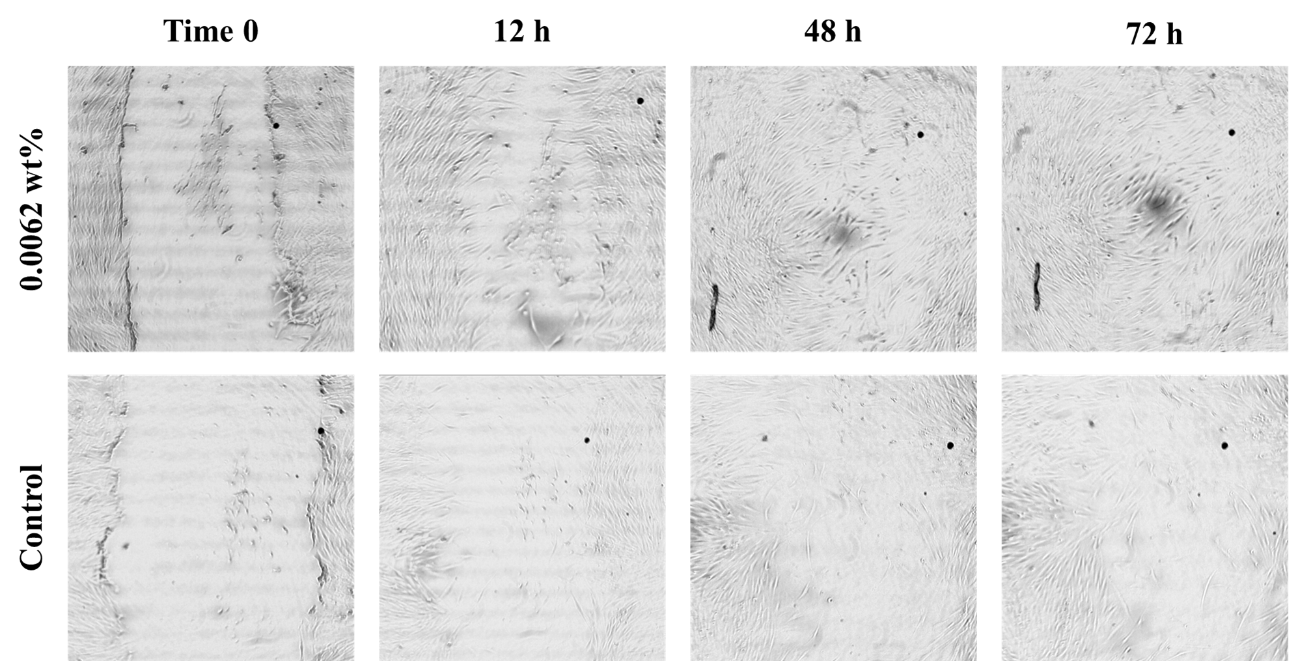


**Figure S5.** Scratch assays of samples incubated in DMEM containing 0.0062 wt% of lipoyl-KTTKS and only with DMEM without serum to evaluate the migration of HDFa cells and simulate wound recovery.

**Table S1**. SANS and SAXS data fitted parameters. Data fitted using SASfit,^[1-2]^ using a long cylindrical shell form factor model, with allowance for monomers via a Generalized Gaussian coil factor for SAXS data.

| **Parameter** | **SANS 3 wt% lipoyl-KTTKS** | **SAXS 3 wt% lipoyl-KTTKS** | **SAXS 3 wt% lipoyl-KTTKS with 2.6 wt% TCEP** |
| --- | --- | --- | --- |
| *R*_o_ / Å | 19.0 ± 22.3 | 25.0 ± 25.0 | - |
| *t* / Å | 9.10 | 10.0 | - |
| *η*_o_ / cm^-1^ | -6.97×10^-7^ | -1.83×10^-7^ | - |
| *η*_s_ / cm^-1^ | 2.16×10^-6^ | -4.11×10^-7^ | - |
| *L* / Å | 800 | 987 | - |
| *I*_1_ / cm^-1^ |  |  | 3.94×10^-7^ |
| *n* |  |  | 2.99 |
| *R*_g_ / Å | - | 22.0 | 6.3 |
| ν | - | 0.30 | 0.35 |
| *I* / cm^-1^ | - | 0.10 | 0.013 |
| *I*_BG_ / cm^-1^ | 0.008 | 0.004 | 0.005 |

Parameters: **Generalized Gaussian Coil:** *R*_g_ radius of gyration, ν Flory exponent, *I* intensity; **Long Cylindrical Shell** **Form Factor:** *R*_o_ outer radius, (Gaussian polydispersity Δ*R*); *t* shell thickness, *η*_o_ core scattering contrast, *η*_s_ shell scattering contrast (solvent scattering constrats *η*_solv_ = 0), *L* length; **Power-Law Slope Form Factor:** *I*_1_*q*^-n^; **Background:** *I*_BG_

**References**

[1] I. Bressler, J. Kohlbrecher, A. F. Thünemann, *J. Appl. Cryst.* **2015**, *48*, 1587-1598.

[2] J. Kohlbrecher, I. Bressler, *J. Appl. Cryst.* **2022**, *55*, 1677-1688.
